# Primary yolk sac tumour of the urinary bladder: A case report and review of the literature

**DOI:** 10.3892/ol.2013.1670

**Published:** 2013-11-08

**Authors:** WING HO MUI, KA CHAI LEE, SIN CHUEN CHIU, CHUN YIN PANG, SAU KWAN CHU, CHI WAI MAN, CHI SING WONG, WING KIN SZE, YUK TUNG

**Affiliations:** 1 Department of Clinical Oncology, Tuen Mun Hospital, Tuen Mun, Hong Kong SAR, P.R. China; 2 Department of Pathology, Tuen Mun Hospital, Tuen Mun, Hong Kong SAR, P.R. China; 3 Department of Surgery, Division of Urology, Tuen Mun Hospital, Tuen Mun, Hong Kong SAR, P.R. China

**Keywords:** yolk sac tumour, urinary bladder, ketamine abuse

## Abstract

We present a case of rare primary yolk sac tumour of the urinary bladder in adulthood. A 31-year-old female patient presented with a history of chronic ketamine abuse, which has not previously been shown to be associated with malignancy development. The final diagnosis was established only after radical cystectomy. A computed tomography (CT) scan showed paraaortic lymph node metastasis. The patient was treated with systemic chemotherapy. A review of the literature revealed that surgical excision and cisplatin-based chemotherapy remain to be the standard of care for extragonadal yolk sac tumours.

## Introduction

There are five types of germ cell tumours according to clinical presentation, pathology and cytogenetics. Type I tumours (teratomas and yolk sac tumours) are more frequent in extragonadal sites than in the gonads; whereas type II tumours (seminomas and non-seminomas) occur mainly in the gonads (1) Primary urinary bladder germ cell tumours are exceedingly rare. The current report presents a case of primary yolk sac tumour of the urinary bladder in a 31-year-old female ex-ketamine abuser. The diagnosis of a yolk sac tumour at this rare site can be difficult and biopsy alone is not reliable. No previous studies have reported a correlation between chronic ketamine abuse and urinary bladder malignancy development. Informed consent was obtained from the patient.

## Case report

In 2007, a 26-year-old female presented to the Department of Urology of the Tuen Mun Hospital (Hong Kong, China) with bilateral hydronephrosis. Since 2000, the patient had been consuming 8–10 ketamine tablets daily from illicit sources. Cystoscopy revealed cystitis and a biopsy showed florid reactive changes in the urinary bladder associated with erosion involving the urothelium, which underwent extensive intestinal metaplastic changes. The patient defaulted follow-up examinations.

**Figure 1:**
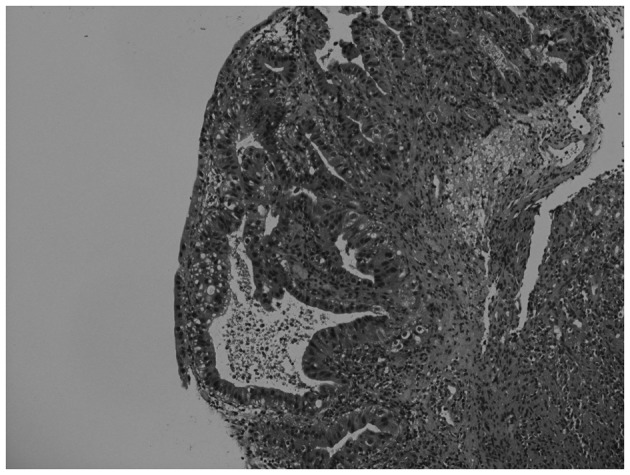
Pathological features indicative of adenocarcinoma (H&E; magnification, ×100).

**Figure 2:**
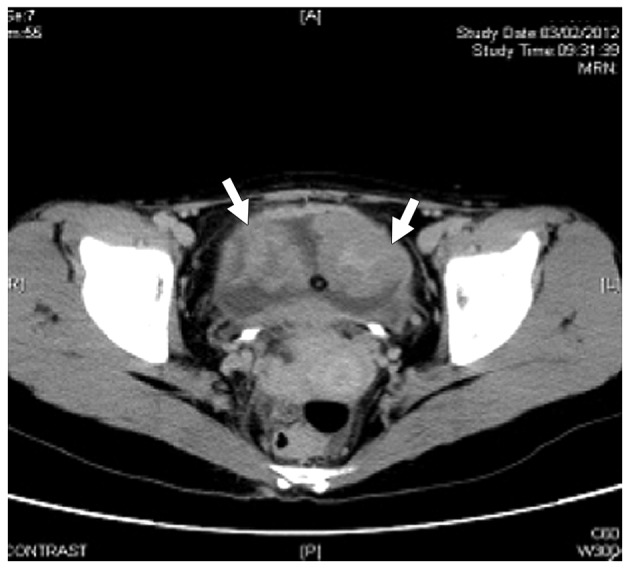
Computed tomography (CT) scan of the abdomen. Arrows indicate urinary bladder masses.

**Figure 3:**
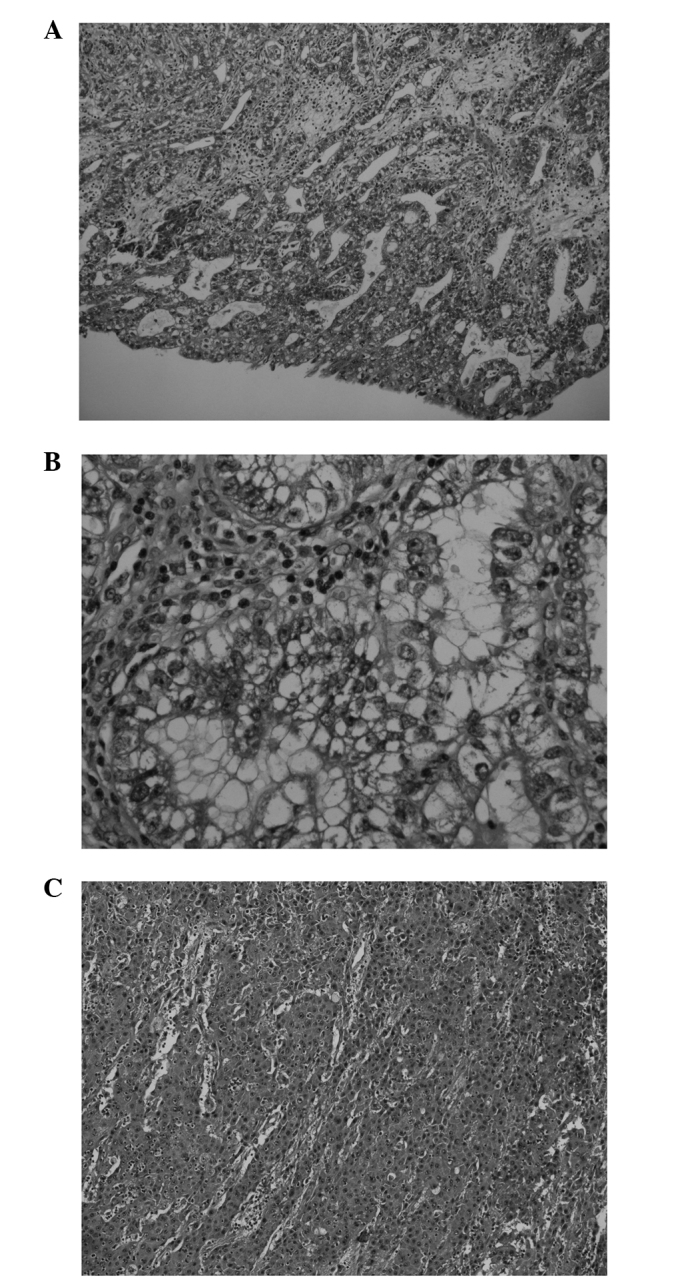
(A) Glandular area (H&E; magnification, ×100). (B) Cytoplasmic hyaline globules in tumour cells (H&E; magnification, ×400). (C) Hepatoid and solid area (H&E; magnification, ×100).

**Figure 4:**
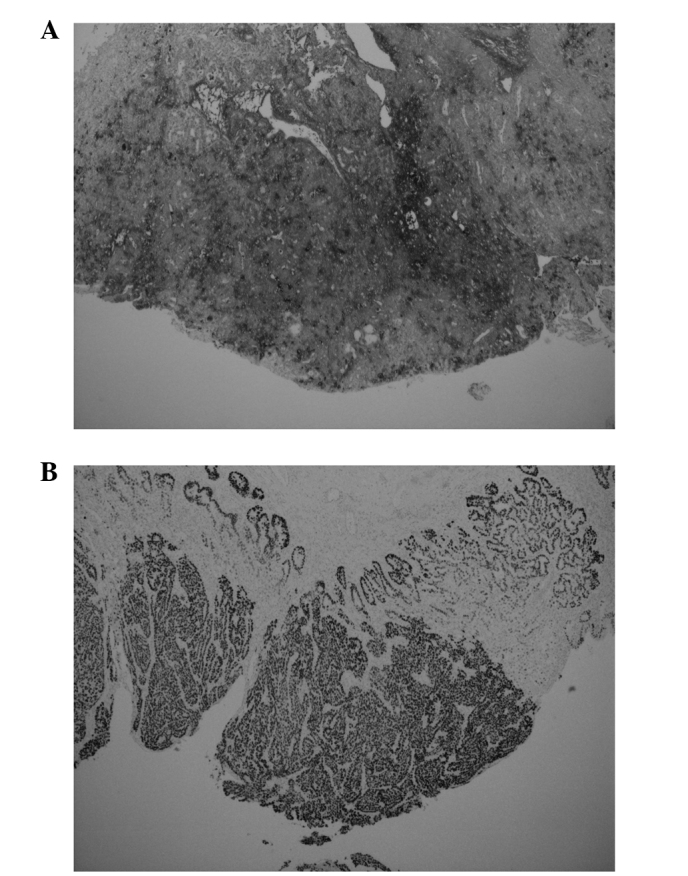
(A) Patchy and moderate reactivity to αFP (magnification, ×40). (B) Diffuse and strong reactivity to SALL4 (magnification, ×40). αFP; α-fetoprotein; SALL4, Sal-like protein 4.

**Figure 5:**
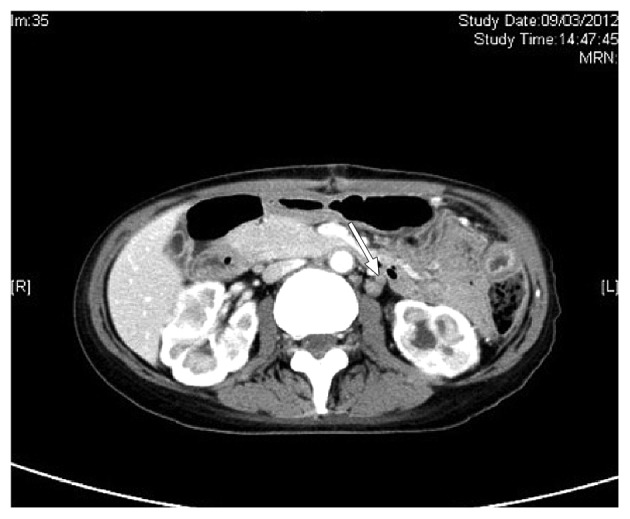
Computed tomography (CT) scan of the abdomen and pelvis. Arrow indicates enlarged paraaortic lymph nodes.

In January 2012, the patient presented again with gross haematuria. Cystoscopy identified a 4-cm whitish mass at the dome of the urinary bladder, with pathological features indicative of adenocarcinoma (Fig. 1). A computed tomography (CT) scan of the abdomen was performed on February 3, 2012, which demonstrated masses within the urinary bladder (Fig. 2). A transurethral resection of the bladder tumours was subsequently performed on January 31, 2012. Pathological examination showed a muscle-invasive, poorly-differentiated carcinoma with a clear cell component. The patient underwent a radical cystectomy and T-pouch orthotopic substitution cystoplasty on February 21, 2012. Two similar 5-cm tumours were present, with various histological morphologies in different areas, featuring reticular, glandular, papillary, microcystic, solid and hepatoid patterns (Fig. 3). The tumour cells showed immunohistochemical reactivity for antibodies against MNF116, α-fetoprotein (αFP) and Sal-like protein 4 (Fig. 4), but not against EMA, HCG, placental alkaline phosphatase, CD30 and octamer-binding transcription factor 3/4. The final pathological diagnosis was of a yolk sac tumour. A CT scan of the abdomen and pelvis on March 9, 2012, demonstrated several enlarged paraaortic lymph nodes of ≤1.5 cm in size (Fig. 5). No sites of suspicious ovarian primary or other disease involvement were identified. An examination performed by a gynaecologist did not reveal a primary gynaecological site of disease.

The serum αFP level was found to be raised (1,028 ng/ml) on March 13, 2012, but fell to 45.9 ng/ml on April 17, 2012, prior to undergoing chemotherapy. In view of the impaired renal function (48.9 ml/min creatinine clearance, according to the Cockcroft-Gault formula), the patient was treated with the combination chemotherapy JEB regimen (day 1, area under curve 5 mg/ml/min carboplatin, intravenously; days 1–5, 100 mg/m^2^ etoposide, intravenously; days 1, 8 and 15, 30 mg bleomycin, intravenously; and the regimen was repeated every 21 days). Two cycles of chemotherapy were administered between April and May 2012. The first cycle of chemotherapy was complicated by grade 3 mucositis and neutropenic sepsis. Moreover, the second cycle was complicated by appendicitis with intra-abdominal abscess formation requiring laparotomy, intensive care and prolonged post-operative management. It was then decided to terminate the chemotherapy. The patient was put on close clinical surveillance, including regular serum tumour marker analysis and CT scan monitoring. The final CT scan, on July 23, 2012, showed no interval change of the paraaortic lymph nodes. The patient’s nadir serum αFP level following chemotherapy was 14.1 ng/ml on August 9, 2012.

## Discussion

Primary germ cell tumours of the urinary bladder are extremely rare. In the present review of the literature, <10 cases had been previously reported (2–7). To the best of our knowledge, the current case is the first reported primary yolk sac tumour of the urinary bladder in adulthood.

The hypotheses for the development of extragonadal germ cell tumours include the following: i) Failure of the primitive germ cells to complete the normal migration along the urogonadal ridges; ii) germ cell tumours undergo reverse migration; iii) germ cell tumours are the metastatic deposits from occult gonadal primaries; and iv) germ cell tumours result from the germ cells distributed to other organs physiologically for function (1,8,9).

The accurate pathological diagnosis of germ cell tumours and the distinction from non-germ cell tumours is critical, as the majority of cases of germ cell tumours are potentially curable, particularly in young patients. Diagnosing a yolk sac tumour may be difficult since a yolk sac tumour may assume a variety of architectural patterns, including microcystic, solid, myxomatous, papillary, polyvesicular vitelline, alveolar, glandular, hepatoid and intestinal (10,11). These may explain the diagnoses of adenocarcinoma and invasive carcinoma in the present patient’s previous pathological examinations. The presence of the particularly characteristic histological Schiller-Duval body, which consists of arrays of neoplastic cells surrounding a central vessel in a glomeruloid appearance, aided the diagnosis (12–14). Beside classical histological features, immunohistochemical study is essential for determining the correct diagnosis. This is based on the relatively specific immunohistochemical profiles carried by the various types of germ cell tumour (Table I) (12,14–18).

The patient’s medical history in 2007 identified a dysplastic process occurring in the urinary bladder. However, in the present review of the literature, the correlation between ketamine abuse and the formation of a urinary bladder malignancy was shown to have not previously been documented. The chronic recreational use of ketamine and its associated urinary system issues has become a global issue (19–21). The new medical entity ‘ketamine-induced ulcerative cystitis’ originated from a publication by Shahani *et al*(21) in 2007. Common symptoms include frequent urination, urge incontinence and painful haematuria. The method of production and composition of illicit ketamine is unclear, and the chemicals and metabolites responsible for the pathogenesis are not well known. Cystoscopic observations may reveal features of cystitis and ulceration, characterised by granulation tissue formation and fibrosis in the epithelium and lamina propria (21). Although there is eosinophilic infiltration, the overall pathology of chemical cystitis is distinct from that of eosinophilic cystitis, which has been reported to be associated with transitional cell carcinoma (22).

Despite their rarity, extragonadal yolk sac tumours mainly affect children and young females (9–11,13). The mediastinum and retroperitoneum are the most common extraovarian primary sites. Less common sites may include the omentum, vagina and brain (10–12,17). These tumours are highly aggressive, harbouring the tendency for early lymphatic and haematological metastasis to distant sites (11,17). Long-term survival rates, specifically for extragonadal yolk sac tumours, are not well known. The International Germ Cell Cancer Collaborative Group data, determined the 5-year survival rate for mediastinal non-seminoma (i.e. poor risk group) as 48% (23), and a large case series from the German group of extragonadal germ cell tumours showed similar survival rates (8,24,25). Extragonadal germ cell tumours have been managed under the same principle as their primary gonadal counterparts, using treatment comprised of systemic chemotherapy together with local treatment*,* including surgery and radiotherapy. Adjunct chemotherapy following local surgical treatment is recommended by the majority of studies if the disease is operable upfront; cisplatin-based regimens are widely used (8–11,13,24–27). Regimens, including bleomycin, etoposide and cisplatin (BEP) and vinblastine, ifosfamide and cisplatin (VIP), are the most commonly adopted. In addition, high-dose chemotherapy followed by autologous bone marrow transplantation is used (8,24,28). To date, the evidence has not been sufficient to determine the optimal chemotherapy duration for extragonadal germ cell tumours, including yolk sac tumours. Four cycles of cisplatin-based combination chemotherapy is the most prevalent treatment regime (8,11,12,24,26,27), and factors taken into account prior to treatment include patient age, performance status, organ function (particularly the lungs and kidneys), histology, serum marker levels, the location of the primary tumour and the sites of the metastases (23,28,29).

In conclusion, the diagnosis of a yolk sac tumour may be challenging in sites of rare occurrence. A high level of clinical suspicion is required, particularly in young patients. Prior to the availability of new therapeutic agents, systemic cisplatin-based chemotherapy remains the standard of care for extragonadal germ cell tumours.

## Figures and Tables

**Table I:** Immunohistochemical study of germ cell tumours.

Type of germ cell tumour	Reactivity
Seminoma/dysgerminoma	PLAP, c-kit and Oct3/4
Spermatocytic seminoma	c-kit and PLAP
Embryonal carcinoma	MNF116 (cytokeratin), CD30, Oct3/4 and SALL4
Yolk sac tumour	αFP and SALL4
Choriocarcinoma	MNF116 (cytokeratin)

PLAP, placental alkaline phosphatase; Oct3/4, octamer-binding transcription factor 3/4; αFP, α-fetoprotein; SALL4, Sal-like protein 4.
